# The Effect of Nutrients on Neurological Disorders

**DOI:** 10.3390/nu16234016

**Published:** 2024-11-23

**Authors:** Lorena Perrone, William B. Grant

**Affiliations:** 1 Department of Medicine and Surgery, University KORE of Enna, 94100 Enna, Italy; 2 Sunlight, Nutrition, and Health Research Center, 1745 Pacific Ave., Suite 504, San Francisco, CA 94109, USA

The prevalence of neurological disorders (NDs) is increasing, with great cost to public health [1]. In addition, the molecular pathways that cause several NDs are not fully understood, leading to delayed diagnosis and therapeutic treatments that treat symptoms instead of targeting the causative molecular alterations [2]. Both epidemiological studies and prospective cohort studies indicate that good nutrition exerts a beneficial effect on health, including its positive role in brain function [3]. Increasing evidence also shows that diet and nutrients play an important role in preventing and managing several NDs. In particular, data suggest that defined dietary patterns are beneficial against NDs, mostly through their anti-inflammatory function [4]. Indeed, data indicate that chronic low-grade inflammation contributes to the progression of several NDs, including psychiatric disorders and neurodegenerative diseases [5,6]. However, foods rich in high saturated fats, trans fats, processed meats, and refined grains and sugar promote an inflammatory response, leading to chronic inflammation, and their consumption correlates with higher risk of NDs [7]. Thus, dietary intervention is a promising therapeutic strategy to prevent and manage NDs. Studies showing how dietary patterns and nutrients affect ND progression, therefore, play an important role in managing diseases with such a high impact on well-being and public health.

This Special Issue, titled “The Effect of Nutrients on Neurological Disorders”, collects original articles illuminating how diet and nutrients affect NDs.

Cognitive impairment is a burdensome disease that strongly affects people’s well-being, as well as having an economic impact on public health. Cognitive impairment affects mostly older people. Owing to higher life expectancy, the number of people suffering from cognitive impairment is rapidly increasing [8]. Several studies showed that environmental factors and lifestyle contribute to the onset of cognitive decline [9]. This Special Issue includes four articles analyzing four aspects of environmental factors that modulate the progression of cognitive decline, including heavy metal exposure, nutritional supplements, and dietary habits.

Several studies indicate that heavy metal exposure affects human health, including cognitive function [10]. Enhanced heavy metal exposure comes from cigarette smoke, air pollution, and the contamination of water and food. Such exposure then contributes to the pathophysiology of cognitive impairment [10]. In this Special Issue, Song and colleagues offer a study analyzing the combined effects of five mixed metals on cognitive function. The researchers also investigate the correlation between a person’s sex and the effects of exposure to five metals on cognitive function [11]. That large cross-sectional cohort study includes 1833 older Americans (883 males and 950 females) and analyzes blood levels of mercury, cadmium, lead, manganese, and selenium in correlation with cognitive performance assessed via four cognitive tests. The study reports that blood levels of lead and cadmium correlate with diminished cognitive performance, whereas selenium blood levels correlate with better cognitive performance. High levels of selenium counteract the other metals’ negative effects on cognitive function [11]. The study also shows the gender specificity of the correlation between the ratio of levels of metals in the blood and cognitive performance [11], suggesting that environmental heavy metals affect cognitive function through sex-specific mechanisms.

Several studies analyze how dietary supplements and nutraceuticals affect cognitive function, suggesting a beneficial effect, proposing them as ways to alleviate the progression of cognitive impairment [12]. In that regard, Leonard and colleagues, in this Special Issue, investigate how ashwagandha supplementation affects levels of biomarkers for cognitive and psychological function [13]. For more than 3000 years, ashwagandha (*Withania somnifera*) has been used as an anti-inflammatory, immunomodulatory, and antioxidant agent to manage mood disorders, traumatic brain diseases, and neurodegenerative diseases [14,15,16,17,18,19]. The researchers carry out a prospective clinical trial on 59 males and 59 females, along with age- and body mass index-matched control subjects, treated with ashwagandha supplementation, with liposomes used as the carrier. Cognitive performance and mood states are compared in treated and control subjects. Ashwagandha supplementation enhances memory and other cognitive functions and improves some mood aspects such as tension and fatigue [13]. Those data indicate that ashwagandha could be a beneficial nutraceutical capable of improving cognitive performance and mood.

The active metabolic form of vitamin D_3_, 1,25-dihydroxyvitamin D_3_ [1,25(OH)_2_D_3_; also called calcitriol], exhibits beneficial effects on various NDs, including cognitive disfunction and Alzheimer’s disease (AD). Calcitriol modulates inflammation, oxidative stress, and energy balance, as well as having a neuroprotective function [20]. Supplementation with vitamin D is proposed as a complementary treatment for various NDs, including AD [21]. To correlate the effect of vitamin D supplementation, researchers can measure two metabolites of vitamin D_3_: blood levels of 1,25(OH)_2_D_3_, the active form, and 25(OH)D_3_, which has a higher blood concentration and is more stable than the active form with its short half-life. Indeed, 25(OH)D_3_ levels remain stable for almost 2 weeks [22]. However, clinical studies analyzing blood levels of 25(OH)D_3_ are hard to compare because the studies are carried out in different laboratories using different assays. Measuring 25(OH)D_3_ requires further standardization because the assays employed in different laboratories yield significant differences [22]. Since 1999, it has been known that prospective cohort studies are subject to the underestimation of the effect of health outcomes due to changes in the variables after baseline. Such underestimation is called “regression dilution” [23]. Grant’s review, in this Special Issue, looks at regression dilution after long follow-up times and shows its effects on results from various prospective cohort clinical studies (nine for all-cause dementia, six for AD, and nine for cognitive impairment), analyzing the relationship between vitamin D deficiency and cognitive disorders [24]. His study shows that in prospective cohort studies, the vitamin D-dependent risk of developing cognitive diseases exhibits an inverse correlation with the mean follow-up period [24]. When the follow-up period is taken into account, the regression fit to the shortest period finds that the association of high vs. low 2(OH)D is about twice as high as that calculated by averaging the findings from all of the studies. Those data are relevant for setting up cohort studies aimed at analyzing how vitamin D affects the risk of developing dementia and cognitive impairment, as well as many other health outcomes.

An increasing number of studies show that the Mediterranean diet (MeDi) and the Mediterranean lifestyle reduce the risk of several chronic diseases, including cognitive impairment and dementia [25,26,27,28,29,30,31]. The MeDi consists of high consumption of vegetables, fruits, legumes, nuts and seeds, whole grains, and olive oil; moderate consumption of fish; and very low consumption of red meat [32]. The MeDi is enriched in natural molecules that benefit health and brain function [33], and the diet shows reduced levels of proinflammatory and pro-oxidant molecules, such as the advanced glycation end-products involved in the progression of several chronic diseases [34]. Dominguez and colleagues present, in this Special Issue, an original study investigating adherence to the MeDi and the level of physical activity in 73 patients affected by mild-to-moderate AD and 73 age-matched control subjects [35]. Their study shows that the dietary pattern of AD patients is one of low adherence to the MeDi; AD patients also had less physical activity than control subjects. By using a multivariate analysis, the researchers show that only AD significantly correlates with adherence to the MeDi, whereas sex, physical activity, polypharmacy, and comorbidities exhibit no correlation with MeDi adherence [35]. The study further shows the value of high adherence to the MeDi to prevent AD.

The review article of Sbai and colleagues, in this Special Issue, summarizes recent data investigating the MeDi’s role in the progression of diabetic retinopathy (DR), age-related macular degeneration (AMD), and glaucoma [36]. Visual impairment adversely affects health, quality of life, and cognitive and psychological development, and the therapies are expensive [37]. This is why researchers have shown increased interest in dietary habits that can prevent retinal diseases. Sbai and colleagues effectively describe the characteristics of the MeDi and the beneficial natural molecules enriched therein. The article underlines the differences among the MeDi scoring systems used to analyze MeDi adherence in cohort studies—variations that cause difficulties in comparing results from different scoring systems [36]. Moreover, the review summarizes the molecular pathways induced by the MeDi, in particular the activity of Nrf2, which counteracts the reactive oxygen species-induced cell damage that characterizes retinal diseases [38]. Finally, their review gives an accurate overview of recent data analyzing the role that MeDi adherence plays in preventing DR, AMD, and glaucoma. The researchers do so by summarizing findings from cohort studies, animal models, and in vitro analysis showing that high MeDi adherence plays a crucial role in preventing those diseases by lowering risk and delaying onset and progression. Moreover, supplements can help as adjuvant therapies in preventing those diseases but cannot be a substitute for the beneficial effects of the MeDi dietary habits and lifestyle [36].

Recent studies show the relevance of the gut microbiome’s modulation of immune system activity and the nervous system. Dysbiosis affects the equilibrium between the gut microbiota and the host, promoting chronic inflammation and contributing to several diseases, including NDs [39,40,41]. Dietary habits and nutrients play an important role in determining the composition of the gut microbiota, ultimately modulating the gut–brain axis [42]. Hrnciarova and colleagues, in this Special Issue, investigate nutritional supplementation’s role in the gut microbiota and its effect on autism spectrum disorder (ASD) in children [43]. One of the most common neurodevelopmental disorders in children, ASD is characterized by psychiatric and behavioral dysfunction. At present, no known biomarkers are diagnostic for the disorder [44]. The molecular mechanisms responsible for ASD remain unknown. However, recent data show that ASD patients exhibit a peculiar composition of the gut microbiota, indicating that altering the microbiota–gut axis may contribute to ASD pathophysiology [45,46]. Because nutritional supplements modulate the gut microbiota, researchers have investigated using nutritional supplements as a way to improve microbiota composition in people with ASD [47]. Hrnciarova and colleagues analyze an interventional clinical study by supplementing children for 3 months (eight patients with ASD and eight placebo-treated control subjects). This pilot study shows that juvenile supplementation modifies the gut microbiota in children with ASD, ameliorating their symptoms [43]. Those results are promising, opening the way for therapeutic interventions aimed at better treating ASD.

Taste influences dietary habits. The gut microbiota modulates the neurosensorial response and taste, thereby influencing dietary habits, and in turn can contribute to obesity [48]. Thus, dietary patterns can substantially affect public health [49]. Obesity is associated with the onset of multiple sclerosis (MS) in children and young people [49]. MS is an autoimmune disease characterized by chronic inflammation and affects the central nervous system [50]. Papetti and colleagues examine, in this Special Issue, how obesity affects the onset of MS in children [51]. This prospective clinical study shows that obesity presages the onset of MS in pediatric patients, suggesting that interventions aimed at blocking the development of childhood obesity, such as nutritional intervention, could delay or prevent MS [51].

With their very low prevalence, rare NDs (RNDs) are poorly investigated. Indeed, only a few RNDs have a clear diagnosis that allows for the use of therapeutic strategies that can address the symptoms [52]. Briglia and colleagues present, in this Special Issue, an innovative review summarizing the results of dietary and nutritional intervention in RNDs [53]. Only a few clinical studies and in vitro studies have investigated the role of diet and nutrients in RNDs. Therefore, their narrative review can help with carrying out further investigations in the field. Their study focuses on Angelman syndrome, Rett syndrome, rare leukodystrophies (Krabbe disease and Pelizaeus–Merzbacher disease), rare epilepsy, rare forms of ataxia, and rare brain tumors. Although the literature includes only a few clinical studies of those diseases, Briglia and colleagues highlight the positive results obtained in preclinical studies, which can serve as the basis for further clinical studies.
